# Carcinoma-Associated Fibroblasts Promote Growth of Sox2-Expressing Breast Cancer Cells

**DOI:** 10.3390/cancers12113435

**Published:** 2020-11-19

**Authors:** Angela Dittmer, Jürgen Dittmer

**Affiliations:** Clinic for Gynecology, University of Halle-Wittenberg, Ernst-Grube-Str. 40, 06120 Halle/Saale, Germany; angela.dittmer@uk-halle.de

**Keywords:** carcinoma-associated fibroblasts, breast cancer, Sox2, STAT3, Bcl-3, AKT

## Abstract

**Simple Summary:**

The tumor microenvironment has a strong impact on the behavior of tumor cells. One major cell type residing in the tumor microenvironment is the carcinoma-associated fibroblast (CAF). We were interested in the effect of CAFs on Sox2 (sex determining region Y (SRY)-box 2), which not only is an essential embryonal stem cell transcription factor, but also plays a role in cancer stem cell activity. We found that long-term exposure of ERα-positive breast cancer cells to the cocktail of CAF-secreted factors strongly increased Sox2 expression involving tumor-related proteins and signaling pathways. However, Sox2 was not only present in those tumor cells that express stem cell markers, but was equally abundant in other tumor cells. By being widely expressed, Sox2 may have functions in non-stem cells. In fact, Sox2 was found to regulate ERα expression, to act anti-apoptotically, to promote cellular growth and to protect cells against the anti-estrogen fulvestrant.

**Abstract:**

CAFs (Carcinoma-associated fibroblasts) play an important role in cancer progression. For instance, they promote resistance to anti-estrogens, such as fulvestrant. Here, we show that, in ERα-positive breast cancer cell lines, the cocktail of factors secreted by CAFs (CAF-CM) induce the expression of the embryonal stem cell transcription factor Sox2 (sex determining region Y (SRY)-box 2). Long-term exposure to CAF-CM was able to give rise to very high Sox2 levels both in the absence and presence of fulvestrant. IL-6 (interleukin-6), a major component of CAF-CM, failed to raise Sox2 expression. In MCF-7 sublines established in the presence of CAF-CM, almost all cells showed Sox2 expression, whereas long-term treatment of T47D cells with CAF-CM resulted in a ~60-fold increase in the proportions of two distinct populations of Sox2 high and low expresser cells. Exposure of BT474 cells to CAF-CM raised the fraction of Sox2 high expresser cells by ~3-fold. Cell sorting based on CD44 and CD24 expression or ALDH (aldehyde dehydrogenase) activity revealed that most Sox2 high expresser cells were not CD44^hi^/CD24^lo^- or ALDH-positive cells suggesting that they were not CSCs (cancer stem cells), though CD44 played a role in Sox2 expression. Functionally, Sox2 was found to protect CAF-CM-treated cells against apoptosis and to allow higher growth activity in the presence of fulvestrant. Mechanistically, the key drivers of Sox2 expression was found to be STAT3 (Signal transducer and activator of transcription 3), Bcl-3 (B-cell lymphoma 3) and the PI3K (Phosphoinositide 3-kinase)/AKT pathway, whose activities/expression can all be upregulated by CAF-CM. These data suggest that CAF-CM induces Sox2 expression in non-CSCs by activating proteins involved in growth control and drug resistance, leading to higher protection against apoptosis.

## 1. Introduction

Breast cancer, the most common type of cancer and leading cause of cancer-associated mortality among women worldwide [1], is a heterogenous disease. The majority of breast cancers is positive for ERα (estrogen receptor α) and can be successfully treated with anti-estrogens, such as tamoxifen or fulvestrant, or with aromatase inhibitors [2]. Her2 (human epidermal receptor 2) is another important driver in breast cancer and can be targeted by a variety of drugs, including anti-Her2 antibody trastuzumab [3]. No selective drugs are available for triple-negative breast cancers, which are devoid of ERα, Her2, and PR (progesterone receptor) and therefore chemotherapy is still the routine way to treat this breast cancer subtype [4].

A major challenge in cancer treatment is the development of drug resistance. Many mechanisms have been described that lead to drug resistance [5,6]. The acquisition of drug resistance is often promoted by the microenvironment [7,8,9]. A subpopulation in breast cancer, which are naturally resistant to many drugs, are CSCs [10]. Not eradicated by treatment they can give rise to minimal residual disease and re-initiate cancer growth, once treatment is omitted [11]. Particularly, quiescent CSCs are likely to survive treatment [12,13]. Previously, quiescent CSCs have been distinguished from proliferating CSCs by the expression of certain markers [14]. While quiescent CSCs are mesenchymal cells that show high expression of CD44 and low levels of CD24, proliferative CSCs are epithelial cells that are positive for certain ALDHs (aldehyde dehydrogenases). The proportions of the two types of CSCs within the tumor cell mass varies depending on the breast cancer subtype and depending on the environmental conditions. In addition, a new concept of CSC hierarchy proposes higher plasticity of tumor cells and claims that recruitment of CSCs from non-CSCs may be more frequent than previously thought [12]. This plasticity is greatly dependent upon the microenvironment. Hence the microenvironment not only plays an important role in drug resistance, but also in the regulation of the CSC pool.

Of the many microenvironmental factors, CAFs are key mediators of microenvironment-induced drug resistance [15] and, as CSC niche cells, can contribute to the maintenance of CSC activity [16]. Among the different types of CAFs is the secretory type, which provide tumor cells with a cocktail of factors that can strongly affect tumor cell activities [17]. We have previously shown that conditioned medium derived from CAFs (CAF-CM) protects ERα-positive breast cancer cells against the anti-estrogen fulvestrant [18]. Furthermore, it increases the expression of some tumor-relevant proteins, such as integrin β1 and Bcl-3 (B-cell lymphoma-3) and the activities of others, such as STAT3 and AKT [18]. Most of these effects could be mimicked by the major CAF-CM component IL-6 (interleukin-6) [19]. Intriguingly, STAT3, AKT, integrin β1 and Bcl-3 are linked to the activities of CSCs or embryonal stem cells [20,21,22,23], while CAF-CM and IL-6 are able to upregulate the RNA level of the CSC marker ALDH1A1 [18,19] and while IL-6 has the potential to increase CSC activity in breast cancer cells [24]. These observations may suggest that CAFs have an impact on CSC activity in breast cancer. Particularly, the embryonal stem cell factor Sox2 may be affected, as this transcription factor can be regulated by the CAF-CM-inducible factors STAT3, AKT, integrin β1, and Bcl-3 [21,25,26,27,28] and can stimulate the expression of ALDH1A1 [19,26]. In support of this hypothesis, it was found that tumor-residing macrophages can induce Sox2 expression in murine breast cancer cells in a STAT3-depending manner and thereby increase CSC activity [29].

There is a large body of evidence that link Sox2 to CSC activity. For instance, Sox2 is involved in mammosphere formation in vitro [30,31,32] and tumor growth in vivo [30,33,34,35]. Sox2 regulates the expression of important CSC-linked genes, such ALDH1A1, LRG5 (leucine rich repeat containing G protein-coupled receptor 5), CD133, and epithelial-to-mesenchymal transcription factors Snail, Slug, and Twist [26,36,37,38,39]. Vice versa, Sox2 is regulated by CSC-relevant proteins, such as CD44, Gli1 (Glioma-associated oncogene homolog 1), Gli2, Six2, and TAZ (transcriptional coactivator with PDZ-binding motif) [31,37,40,41,42]. Lastly, Sox2 is implicated in drug resistance [25,43,44,45,46]. In accordance with its CSC-promoting effects, Sox2 has been linked to worse prognosis in breast cancer [43,47].

Altogether, this prompted us to study the impact of CAF-CM on Sox2 expression and to analyze the potential role of Sox2 in CAF-CM-induced drug resistance. We show here that CAF-CM treatment induces Sox2 expression, whereby long-term exposure can lead to permanently very high Sox2 expression levels. Evidence is presented that Sox2 induces drug resistance by preventing apoptosis. Furthermore, the results suggest that CAF-CM increases the fraction of a subset of cells that heavily express Sox2.

## 2. Results

### 2.1. Long-Term Exposure to CAF-CM Results in Permanently High Expression of Sox2 in Breast Cancer Cells

**Figure 1:**
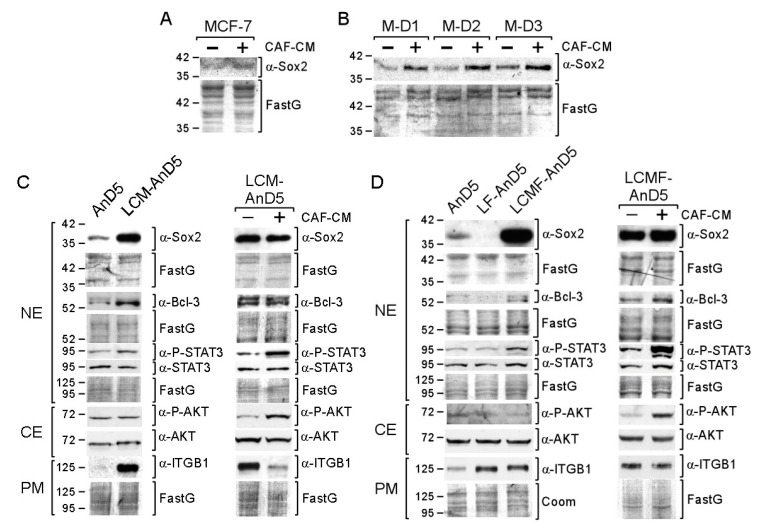
Short- and long-term exposure of CAF-CM (carcinoma-associated fibroblast-conditioned medium) increases Sox2 expression in breast cancer cells. (**A**,**B**) MCF-7 parental or MCF-7/AnD3 subline AnD3/M-D1, AnD3/M-D2, and AnD3/M-D3) cells were grown in the presence or absence of CAF-CM for three days, before nuclear protein extracts were examined for Sox2 expression by Western blot analysis. ((**C**,**D**), left panels) Western blot analyses of AnD5, LCM-AnD5, LF-AnD5 and LCMF-AnD5 cells for the proteins as indicated. Before protein extraction, cells were grown in the absence of CAF-CM and fulvestrant for three days. ((**C**,**D**), right panels) Effect of three-day-treatment of LCM-AnD5 and LCMF-AnD5 cells with CAF-CM on the expression of proteins as indicated. (**C**,**D**) NE = nuclear extract, CE = cytosolic extract, PM = plasma membrane extract, ITGB1 = integrin β1. (**A**–**D**) Protein stain Fast green (FastG) was used to confirm equal protein loading.

We first studied the short-term effect of CAF-CM on Sox2 protein expression. By using Western blot analysis, we examined the abundance of Sox2 in the nuclear protein fraction, where this protein was exclusively found (Figure S1A). As shown for the MCF-7 cell line and the MCF-7 sublines AnD3/M-D1, AnD3/M-D2, and AnD3/M-D3 (Table 1) [48], three-day-exposure to CAF-CM increased Sox2 protein expression (Figure 1A,B).

To study the long-term effects of CAF-CM on Sox2 expression, we exposed the MCF-7 subline AnD5 [48] to CAF-CM for ten weeks to generate the subline LCM-AnD5 (Table 1). Before proteins were isolated for Western blot analysis, cells were either grown in the absence of CAF-CM for three days or further cultured in the presence of CAF-CM. Even in the absence of CAF-CM, the LCM-AnD5 subline showed a much higher Sox2 level than the parental AnD5 subline (Figure 1C, left panel). Re-addition of CAF-CM did not result in a further increase of the Sox2 level (Figure 1C, right panel). These data indicate that long-term CAF-CM treatment can lead to very high levels of Sox2 and that, once this high level is established, CAF-CM is no longer needed. The latter finding might be explained by an adaption of the cells to CAF-CM in a way that they lost general responsiveness to CAF-CM. However, CAF-CM strongly upregulated P-STAT3 and P-AKT levels in LCM-AnD5 (Figure 1C, right panel) showing that these cells are still able to respond to CAF-CM.

To further support these findings, we established two additional sublines, LCMF-AnD5 and LF-AnD5, which were either treated with CAF-CM plus fulvestrant (LCMF-AnD5) or with fulvestrant alone (LF-AnD5) for ten weeks. Fulvestrant was added because Sox2 has been found to be associated with anti-estrogen resistance [43]. To slow, but not to prevent growth of AnD5 cells, the concentration of fulvestrant was adjusted to 1 nM [48]. Like the LCM-AnD5 subline, the LCMF-AnD5 subline showed high Sox2 expression even in the absence of CAF-CM (Figure 1D, left panel). Re-addition of CAF-CM resulted only in a slight increase in the Sox2 level, while it strongly raised P-STAT3 and P-AKT levels (Figure 1D, right panel). The Sox2 level of the LCMF-AnD5 subline was much higher than that of the AnD5 (Figure 1D, left panel) and even higher than that of the LCM-AnD5 subline (Figure S1B). In contrast, LF-AnD5 cells expressed less Sox2 protein than AnD5 cells (Figure 1D, left panel). These data confirm that long-term treatment of CAF-CM strongly upregulates Sox2 expression, whose maintenance do no longer require the presence of CAF-CM. In addition, it seems that fulvestrant enhances the stimulatory CAF-CM effect on the Sox2 expression without having an upregulating effect alone.

Additional support of the long-term effect of CAF-CM on Sox2 was obtained by comparing the Sox2 levels of sublines that we have previously generated (Table 1) [48]. One set of sublines comprised fulvestrant-resistant MCF-7 sublines which were either established in presence of CAF-CM (C-FR1, C-FR2 and C-FR3) or in the presence of MCF7-CM (M-FR1 and M-FR2). Another set of sublines comprised of sublines that were generated from MCF-7/AnD3 cells, that were initially forced to dormancy by high concentration of fulvestrant (1 µM) either in the presence of CAF-CM or in presence of MCF7-CM and, then, after withdrawal of fulvestrant, propagated to become sublines C-D1 and C-D2 or M-D1, M-D2, and M-D3, respectively. In the absence of CM, the CAF-CM-treated sublines in both sets showed higher Sox2 levels than their control sublines (Figure S1C). As shown for C-FR1, re-addition of CAF-CM had no effect on Sox2 expression, while P-STAT3 and P-AKT levels were increased (Figure S1D). Hence, again long-term treatment of CAF-CM resulted in high Sox2 expression, which sustained even when CAF-CM was withdrawn. These data suggest that long-term CAF-CM treatment results in permanently high expression of Sox2 in MCF-7-derived sublines.

IL-6, a major component of CAF-CM, is capable of mimicking many effects of CAF-CM on protein expression [19]. We therefore generated AnD5 sublines that received long-term treatment with rh (recombinant human) IL-6. Since concentrations of ≥5 ng/mL rhIL-6 were found to be toxic to AnD5 cells, when given for several weeks, we used 1.0 and 2.5 ng/mL rhIL-6 to establish the sublines LIL6A-AnD5 and LIL6B-AnD5, respectively. Of note, 2.5 ng/mL rhIL-6 equals the IL-6 concentration to which cells are exposed when the medium contains 20% CAF-CM (data not shown). Irrespective of whether rhIL-6 was present or not, LIL6A-AnD5 showed the same and LIL6B-AnD5 cells even lower Sox2 expression than AnD5 cells (Figure S1E) suggesting that IL-6 was not mediating the upregulatory long-term effect of CAF-CM on Sox2 expression.

### 2.2. Bcl-3, STAT3, and PI3K/AKT Are Involved in CAF-CM-Induced Upregulation of Sox2 Expression

In search of the factors responsible for the high Sox2 levels in the CAF-CM-treated sublines, we noticed that, along with the Sox2 level, Bcl-3, and P-STAT3 levels were elevated in LCM-AnD5 and LCMF-AnD5 cells and, additionally, integrin β1 in LCM-AnD5 cells (Figure 1C,D, left panels). The same three factors showed increased levels also in the CAF-CM-treated fulvestrant-resistant sublines C-FR1, C-FR2, and C-FR3 [48]. In contrast, in LIL6A-AnD5 and LIL6B-AnD5 cells, which do not express Sox2 at high levels, only the Bcl-3 level was upregulated, while the P-STAT3 and integrin β1 levels were similar or lower than those in AnD5 cells (Figure S1E). All three factors have been linked to Sox2 expression, whereby STAT3 is a direct regulator of Sox2 transcription and may be of particular importance [23,27,28,29].

**Figure 2:**
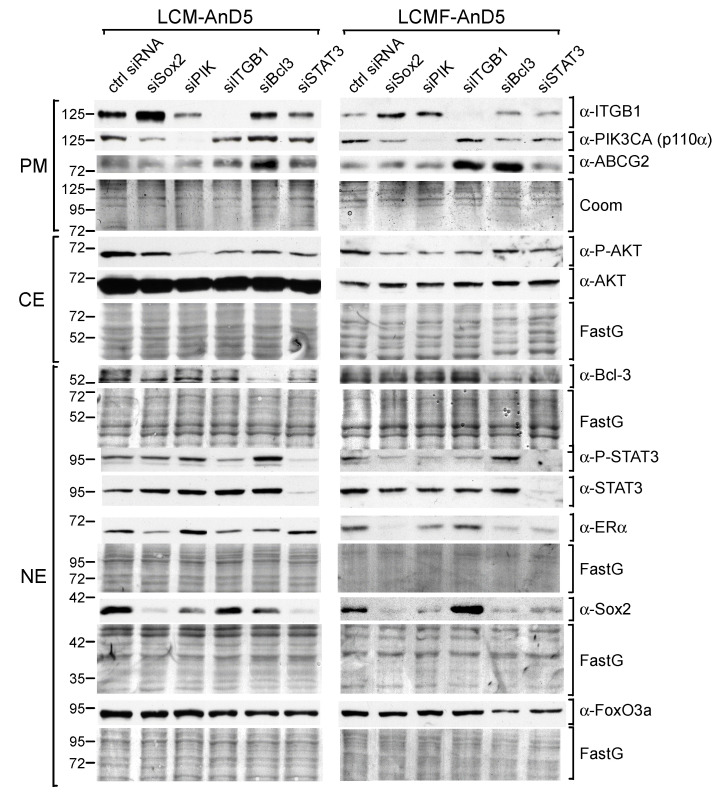
STAT3, Bcl-3, and the PI3K/AKT pathway are key drivers of Sox2 expression CAF-CM- treated sublines. Western blot analyses of protein extracts prepared from the plasma membrane (PM), cytosolic (CE) or nuclear (NE) fraction of LCM-AnD5 and LCMF-AnD5 cells after siRNA transfection and incubation in CAF-CM-free medium for three days. Protein stains Fast green (FastG) or Coomassie (Coom) were used to confirm equal protein loading of nuclear and cytosolic extracts or plasma membrane extracts, respectively. ITGB1 = integrin β1.

To analyze the importance of these factors for Sox2 expression, we treated LCM-AnD5 and LCMF-AnD5 cells with the siRNAs siBcl3, siSTAT3, and siITGB1, which have previously been shown to efficiently knock down their corresponding targets [18,19,48]. We also examined the effect of siPIK, an siRNA which downregulates the PI3K component p110α (encoded by PIK3CA), resulting in dephoshorylation of AKT [19]. AKT, which is activated by CAF-CM (Figure 1C,D, right panels), is also able to regulate Sox2 expression [21,27]. Furthermore, we used siSox2, which potently reduces Sox2 expression (Figure S1F), to examine its effects on the levels of Bcl-3, P-STAT3, P-AKT, and integrin β1 and additionally on the levels of ERα, ABCG2, and FoxO3a, which are potential targets of Sox2 [35,44,49]. The RNA interference results showed that Bcl-3, STAT3 and PI3K/AKT play a critical role in Sox2 expression in both LCM-AnD5 and LCMF-AnD5 cells, though STAT3 seems to be more important in LCM-AnD5, while Bcl-3 and PI3K/AKT play a greater role in LCMF-AnD5 (Figure 2).

Different to siBcl-3, siSTAT3, and siPIK, siITGB1 had either no effect (LCM-AnD5) or even an upregulatory effect on Sox2 expression (LCMF-AnD5). Given that, in LCMF-AnD5 cells, siITGB1 caused downregulation of STAT3 and AKT activity, this result is puzzling. We suspect that, besides the reduction of STAT3 and AKT activities, knock-down of integrin β1 causes additional effects.

Knock-down of Sox2 had three common effects on both sublines (Figure 2). First, it moderately downregulated the p110α level, leading to a slight reduction in the P-AKT level, which is in line with a previous study on nasopharyngeal carcinoma showing that Sox2 can activate AKT by inducing the transcription of the PIK3CA gene [50]. Second, siSox2 strongly downregulated the ERα level in LCMF-AnD5. In addition, other siRNAs that reduced Sox2 expression also downregulated ERα expression. In contrast, in LCM-AnD5 cells, the effect of siSox2 on ERα expression was much weaker. Third, siSox2 raised the expression of integrin β1, which is interesting in view of the finding that siITGB1 increased Sox2 expression in LCMF-AnD5 cells suggesting a negative feedback reaction between Sox2 and integrin β1 in these cells. Additionally, siSox2 reduced the P-STAT3 level in LCMF-AnD5 cells and decreased the Bcl-3 level in LCM-AnD5 cells. No effects of siSox2 could be found on the expression of ABCG2 and FoxO3A.

Collectively, these data suggest that Bcl-3, STAT3, and AKT are involved in maintaining the high Sox2 expression found after long-term exposure to CAF-CM. The results further imply that Sox2 positively feeds back to these same factors, which may further fuel Sox2 expression.

### 2.3. Long-Term Treatment with CAF-CM Increases the Expression of the Stemness Factor Sox2 in the Majority of Cells

**Figure 3:**
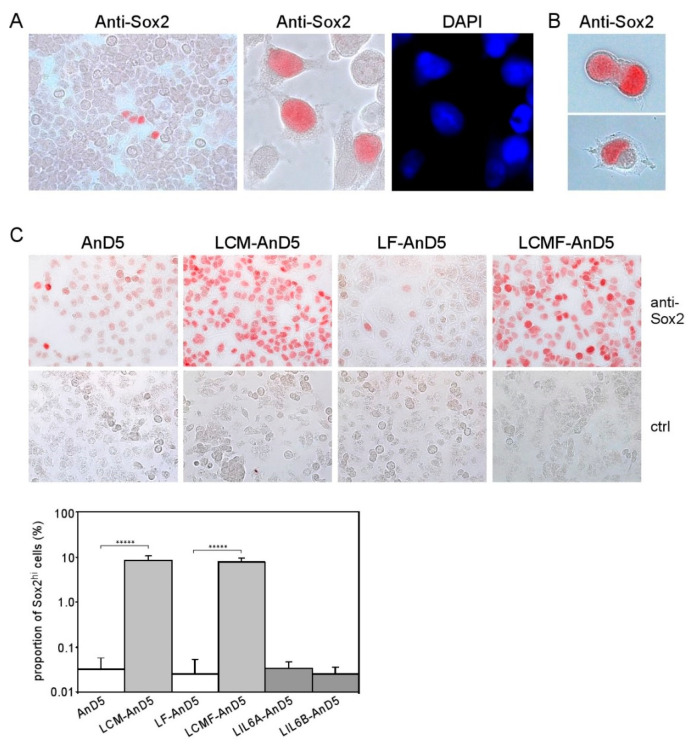
CAF-CM induces Sox2 expression in almost all AnD5 cells. (**A**) Cluster of Sox2-expressing cells in the MCF-7 cell line as detected by immunocytochemistry (ICC), (**B**) Dividing LCMF-AnD5 cells with nuclei which differ in their reactivity to the anti-Sox2 antibody. (**C**) ICC analysis of AnD5, LCM-AnD5, LF-AnD5, and LCMF-AnD5 cells for Sox2 (upper panel) and estimations of the proportions of the Sox2^hi^ cell population (lower panel), also including estimations for LIL6A-AnD5 and LIL6B-AnD5 cells. For each condition, 20 randomly chosen fields/slide were viewed (100–150 cells/field) and the average proportion of the Sox2^hi^ cell population was calculated. The average proportions obtained with three different slides were compared. Statistically analysis was performed by the student’s *t*-test for each matching pair (LCM-AnD5 vs. AnD5 and LCMF-AnD5 vs. LF-AnD5). The asterisks indicate statistical significance (*p* < 0.0001).

Sox2 has been connected to stemness in embryonal development and in cancer [51]. A link to stemness also applies for Bcl-3, STAT3, and AKT. For instance, Sox2 and Bcl-3 cooperate to maintain stemness of embryonal stem cells [23], STAT3 can convert non-CSCs to CSCs [20], AKT is involved in maintaining Sox2-dependent cervical CSC activity [21] and CSCs isolated from ERα-positive cancer show a hyperactive PI3K pathway [22]. Hence, Sox2, Bcl-3, STAT3, and AKT might cooperate to enhance stemness activity. To examine whether Sox2 expression was expressed by all cells or was rather restricted to a minor cell population, we analyzed Sox2 expression by immunocytochemical analysis (ICC). We found that a small percentage of MCF-7 cells showed strong nuclear staining for Sox2 (from now on called Sox2^hi^ cells), while all other cells were negative or weakly positive for Sox2 (Figure 3A). Often Sox2^hi^ cells appeared in clusters of four or more cells. Some cells contained two nuclei, of which one stained heavily for Sox2 and the other showed no or little anti-Sox2 reactivity (Figure 3B). This is reminiscent of asymmetric division, a typical feature of stem cells. We estimated the proportion of Sox2^hi^ cells in the MCF-7 cell line to be ~0.1%, which compares to <1% of ALDEFLUOR-positive CSCs in MCF-7 cells [52,53] and 0.1–1.2% of CD44^+^/CD24^−^/EpCAM^+^-CSCs in ERα-positive cell lines [54]. Hence, the Sox2^hi^ cells in the MCF-7 cell line may be CSCs.

To examine if, in LCM-AnD5 and LCMF-AnD5 sublines, the proportion of Sox2^hi^ cells was increased, we compared Sox2 expression between LCM-AnD5 and AnD5 and between LCMF-AnD5 and LF-AnD5 sublines by ICC. Like the parental MCF-7 cell line, AnD5 and LF-AnD5 sublines contained a Sox2^hi^ cell population at a proportion of ~0.03% (Figure 3C). In addition, in the AnD5, but not in the LF-AnD5 subline, most of the cells displayed a weak positivity for Sox2. This is consistent with the Western blot data showing higher Sox2 expression in the AnD5 compared to the LF-AnD5 subline (Figure 1D, left panel). In the LCM-AnD5 and LCMF-AnD5 sublines, Sox2 expression was increased in two ways: almost all cells expressed much higher amounts of Sox2 than AnD5 cells and the Sox2^hi^ cell population increased to ~8% (Figure 3C). Hence, long-term treatment with CAF-CM strongly increased the expression of Sox2 in almost all cells and raised the proportion of the Sox2^hi^ cell population by ~250-fold. In contrast, LIL6A-AnD5 and LIL6B-AnD5 sublines showed similar Sox2 expression patterns as AnD5 (Figure 3C).

**Figure 4:**
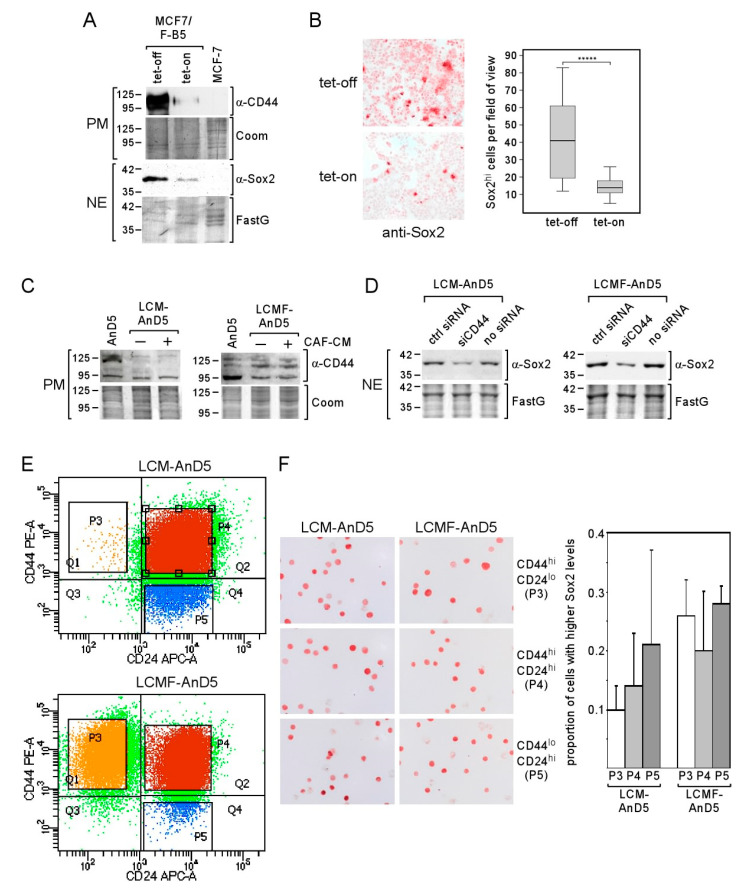
CD44 regulates Sox2 expression, yet Sox2^hi^ cells are not enriched in the CD44^hi^/CD24^lo^ cells population. (**A**,**B**) Tet-regulated CD44 expression in the MCF-7/F-B5 subline and its effect on Sox2 expression as determined by Western blot (**A**) or ICC analysis (**B**). (**B**, right panel) The box-plot shows the differences in the fraction of Sox2^hi^ cells in the tet-on and tet-off modes after one day of incubation. Sox2^hi^ cells were counted in randomly chosen fields of view. For each condition, 40 fields were examined (~200 cells/field). Statistical analysis was performed by using the Mann-Whitney-U-test. The asterisks indicate statistical significance (*p* < 0.0001). (**C**,**D**) CD44 expression in AnD5, LCM-AnD5 and LCMF-AnD5 cells in the presence or absence of CAF-CM (**C**) and the effect of CD44 knock-down on Sox2 expression in LCM-AnD5 and LCMF-AnD5 cells (**D**), as determined by Western blot analysis (PM = plasma membrane extract, NE = nuclear extract, FastG = Fast green). (**E**) Scatterplots of FACS profiles of LCM-AnD5 and LCMF-AnD5 cells after treatment with PE-anti-CD44 and APC-anti-CD24 antibodies. (**F**) Sox2-specific ICC of separated LCM-AnD5 and LCMF-AnD5 cell populations as indicated (left panel). For calculating the proportion of the Sox2^hi^ cell fractions in the three separated cell populations (right panel), P3, P4, and P5 cell fractions were separately sprayed on three slides each. For each slide, the proportion of the Sox2^hi^ population was determined by counting cells in 20 randomly chosen fields (10–20 cells/field). The average proportion of the Sox2^hi^ population in the P3, P4, and P5 fractions were compared. Statistical analysis was done with one-way ANOVA, followed by Bonferroni correction for post-hoc analysis. No statistically significant differences were found.

To clarify if the Sox2^hi^ cell population in LCM-AnD5 and LCMF-AnD5 sublines represent CSCs, we first analyzed the effect of the CSC-related protein CD44 on Sox2 expression. The rationale behind this study was a previous report demonstrating that CD44 can raise Sox2 expression in prostate cancer cells [37]. To examine the importance of CD44 for Sox2 expression in MCF-7 cells, we used the MCF-7 subline MCF7F-B5, which contains a tet (tetracycline)-regulated CD44s expression system [55]. In the tet-off modus, in which CD44 is highly expressed, also Sox2 was substantially upregulated (Figure 4A). This was accompanied by a ~3-fold increase in the proportion of Sox2^hi^ cells and, additionally, by a low Sox2 expression in all other cells (Figure 4B). This prompted us to examine whether CD44 plays a role in Sox2 expression in LCM-AnD5 and LCMF-AnD5 cells. First, we checked overall CD44 expression by Western blot analysis. Compared to AnD5 cells, LCM-AnD5 cells express the same level of the ~95 kD CD44 isotype, but less of the ~110 kD and ~125 kD CD44 isotypes, whereas LCMF-AnD5 cells show a lower level of the ~95 kD isotype, but a higher level of the ~110 kD isotype (Figure 4C). Hence, only LCMF-AnD5 cells show a higher expression at least of one of the CD44 isotypes. Nevertheless, knock-down of CD44 expression by siCD44, which reduced CD44 expression (Figure S1G), decreased the expression of Sox2 in both sublines (Figure 4D). This suggests that Sox2 expression in the LCM-AnD5 and LCMF-AnD5 sublines may be higher in CD44-positive cells.

Encouraged by the data that CD44 plays a role in Sox2 expression in LCM-AnD5 and LCMF-AnD5, we separated cells based on CD44 and CD24 by FACS. We found that the majority of cells were double positive for CD44 and CD24 in both sublines (Figure 4E, subpopulation P4). The proportions of the CD44^hi^/CD24^lo^ subpopulation (P3) were ~0.4% in LCM-AnD5 and ~19% in LCMF-AnD5, while the proportion of CD44^lo^/CD24^hi^ cells (P5) was ~9% and ~1.1%, respectively (Figure 4E). Given these numbers compared to the proportion of the Sox2^hi^ cell population of ~8% in both sublines (Figure 3B), it became clear that, in the LCM-AnD5 subline, Sox2^hi^ cells are unlikely to represent only CD44^hi^/CD24^lo^-CSCs. This notion was supported by the finding that the number of Sox2^hi^ cells was not higher in the CD44^hi^/CD24^lo^ fraction than in the CD44^hi^/CD24^hi^ or in the CD44^lo^/CD24^hi^ fraction derived from LCM-AnD5 cells (Figure 4F). Of note, the fraction of Sox2^hi^ cells seems in general to be higher, when cells are rounded up. Though the proportion of the Sox2^hi^ cell subpopulation (~8%) and that of the CD44^hi^/CD24^lo^ subpopulation (~19%) were similar in the LCMF-AnD5, Sox2^hi^ cells were found in all three fractions at a similar percentage. Hence, in both sublines, Sox2^hi^ cells do not represent CD44^hi^/CD24^lo^-CSCs.

**Figure 5:**
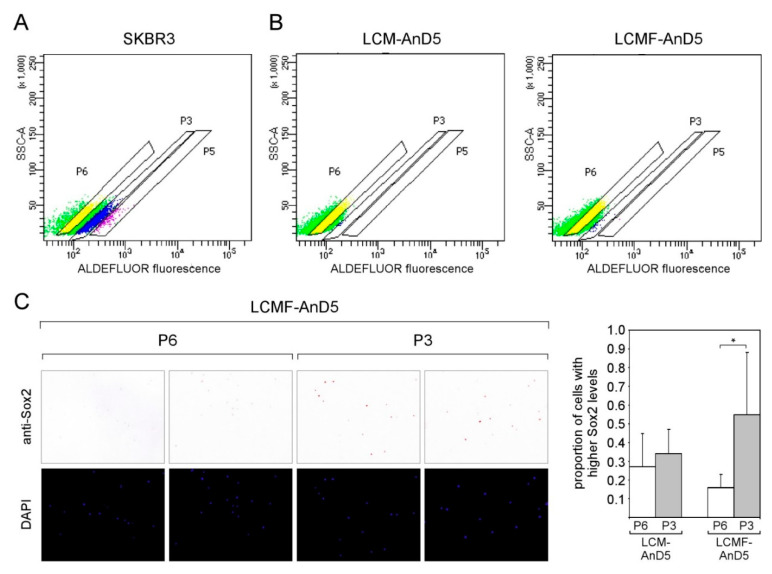
In the LCMF-AnD5 subline, the population of aldehyde dehydrogenase (ALDH)-positive cells contain a higher number of Sox2^hi^ cells. (**A**,**B**) Scatterplots of FACS profiles of SKBR3 (**A**) as well as of LCM-AnD5 and LCMF-AnD5 cells treated with the ALDH substrate BAAA. P3 and P5 populations represent cells with weak and strong ALDEFLUOR fluorescence, respectively, while cells in the P6 population show background fluorescence. Background fluorescence was defined in the presence of the ALDH inhibitor DEAB. ((**C**), left panel) ICC analysis for Sox2 expression. For comparison, cells were stained with DAPI. ((**C**), right panel) Box plot comparing the proportions of cells with higher Sox2 expression in the P3 and P6 populations for LCM-AnD5 and LCMF-AnD5. Cells of the P3 and P6 populations were sprayed on three slides each. Per slide, 20 randomly chosen fields (~20 cells/field) were viewed and the proportion of the Sox2^hi^ cell population was calculated from all 20 fields combined. The average proportion obtained with the three P3 slides were compared with that obtained form the three P6 slides. Statistically analysis was performed by the student’s *t*-test. The asterisk indicates statistical significance (*p* < 0.05).

We next explored the possibility that the Sox2^hi^ cells in LCM-AnD5 and LCMF-AnD5 cells are ALDH-positive CSCs by using the ALDEFLUOR assay. Starting with SKBR3 cells as a positive control we defined three subpopulations P6 (no activity), P3 (weak activity), and P5 (strong activity) (Figure 5A). The proportion of P3 was ~16%, that of P5 ~2%. P3 and P5 combined (~18%), which compares to 15% ALDEFLUOR-positive cells as previously reported for this cell line [56]. Performing the same experiment with LCM-AnD5 and LCMF-AnD5 revealed that a P5 population did not exist and that the proportions of P3 was 0.2% and 0.5%, respectively (Figure 5B). Hence, again, these numbers do not match with the 8% proportion of the Sox2^hi^ cell population in these sublines. Additionally, no difference in the number of Sox2^hi^ cells between the P3 and P5 subpopulations was found with the LCM-AnD5 subline. Hence, Sox2^hi^ cells are not restricted to the ALDH-positive cell subpopulation. Nevertheless, in the LCMF-AnD5 subline, P3 contained ~3-fold more Sox2^hi^ cells than P6 (Figure 5C). These data show that the bulk of Sox2^hi^ cells do not represent ALDH-positive CSCs either, though a small fraction of them may, at least in the LCMF-AnD5 subline.

In sum, these results suggest that long-term CAF-CM treatment of MCF-7/AnD5 cells raised Sox2 expression in almost all cells, whereby a minor fraction of ~8% express Sox2 at a higher level than the majority of the cells. However, to the most part, this minor fraction does not represent CSCs.

### 2.4. CAF-CM-Induced Expression of Sox2 Leads to Better Protection against Apoptosis and Improves Survival in the Presence of the Anti-Estrogen Fulvestrant

**Figure 6:**
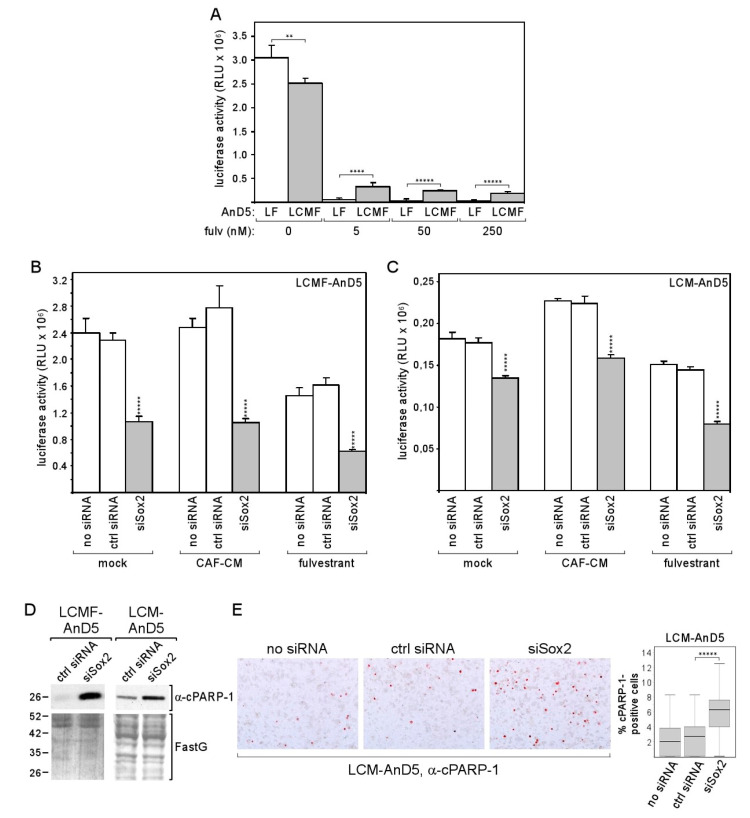
Knock-down of Sox2 increases apoptotic activity of breast cancer cells and reduces their growth activity. (**A**) Comparison of LF-AnD5 and LCMF-AnD5 growth activities by an ATP-based growth assay. Cells were incubated with 0, 5, 50, or 250 nM fulvestrant for 7 days. (**B**,**C**) Effect of siSox2, control siRNA (ctrl siRNA) or mock transfection (no siRNA) on growth activity on LCMF-AnD5 and LCM-AnD5 cells. After transfection cells were grown in the presence of CAF-CM, 1 nM fulvestrant or in the absence of either agent (mock) for 4 days. (**A**–**C**) Each bar represents the average value of five experiments. Statistical analysis was performed with one-way ANOVA, followed by Bonferroni correction for post-hoc analysis. Asterisks indicate statistical significance: ** (*p* < 0.01), **** (*p* < 0.001), ***** (*p* < 0.0001). (**D**) Western blot analysis of cytosolic protein extracts from LCMF-AnD5 and LCM-AnD5 cells for cPARP-1 abundance after transfection with siSox2 or crtl siRNA. FastG (Fast green) staining was used to check for equal protein loading. (**E**) ICC analysis to examine the percentage of cPARP-1-positive cells (left panel). Box plot (right panel) shows the percentage of cPARP-1-positive cells in the presence of siSox2, crtl siRNA, or in the absence of any siRNA. For each condition, 40 fields of view (~200 cells/field) were examined. Statistical analysis was performed by using the Mann-Whitney-U-test. The asterisks indicate statistical significance (*p* < 0.0001).

We next explored the possible benefit of the Sox2 expression in LCM-AnD5 and LCMF-AnD5 cells. Given the evidence that Sox2 is linked to the resistance to the anti-estrogen tamoxifen [43], we compared the effect of fulvestrant on growth of the LCMF-AnD5 and LF-AnD5 sublines, which, as mentioned above, were both adapted to 1 nM fulvestrant and were established side-by-side. We found that the LCMF-AnD5 subline was much more resistant to fulvestrant at higher concentrations (5, 50 and 250 nM) than its control subline LF-AnD5 (Figure 6A). By transfecting cells with siSox2, we could confirm that Sox2 is important for growth in the presence of fulvestrant (Figure 6B). However, siSox2 similarly reduced growth also in the absence of fulvestrant suggesting that Sox2 is generally important for cellular growth. Data obtained with the LCM-AnD5 subline confirmed this notion, though the effects of siSox2 were generally weaker (Figure 6C). Other studies that employed blockage of Sox2 gene activation or shSox2 to inhibit Sox2 expression also showed that Sox2 was important for the growth activity of breast cancer cells [34,56]. The growth-promoting effect of Sox2 may have to do with its potential anti-apoptotic activity, which has been previously reported [56,57]. In support of this notion, we found an increased abundance of the apoptosis-indicating 25kD PARP-1 (poly [ADP-ribose] polymerase 1)-fragment cPARP-1 (cleaved PARP-1) in the LCM-AnD5 and LCMF-AnD5 sublines after treatment with siSox2 (Figure 6D), whereby cPARP-1 abundance correlated with the ability of siSox to reduce growth, which were both higher in LCMF-AnD5 cells. The latter suggest that the pro-apoptotic activity of siSox2 was the major reason for its growth-inhibitory effect. To further confirm the anti-apoptotic activity of Sox2, we performed ICC with LCM-AnD5 cells and compared the number of cells that stain positive of cPARP-1 in the presence and absence of siSox2. In the presence of siSox2, the number of cPARP-1-positive cells were ~3-fold higher than under control conditions (Figure 6E).

The data imply that, by upregulating Sox2 expression, CAF-CM improves protection against apoptosis and allows a higher survival rate in the presence of the anti-estrogen fulvestrant.

### 2.5. Long-Term CAF-CM Treatment Increases Sox2 Expression Also in Other ERα-Positive Breast Cancer Cell Lines

**Figure 7:**
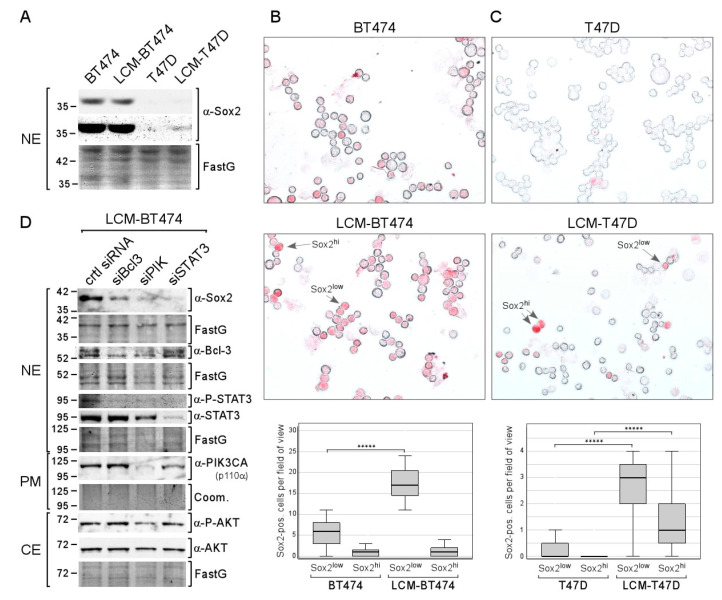
Long-term treatment with CAF-CM increases the number of Sox2-expressing cells in the T47D and BT474 breast cancer cell lines. (**A**) Western blot analysis of nuclear extracts derived from BT474, LCM-BT474, T47D, and LCM-T47D cells for Sox2 expression. (**B**,**C**) ICC analyses of the BT474 and LCM-BT474 (**B**) as well as the T47D cells and LCM-T47D cell lines (**C**) for Sox2-expressing cells. Examples of Sox2^hi^ cells and Sox2^low^ cells are indicated by arrows. Box plots show the number of Sox2^hi^ cells or Sox2^low^ cells per field of view (50–100 cells/field). For each condition, 40 fields were examined. Statistical analysis was performed by using the Mann-Whitney-U-test. The asterisks indicate statistical significance (*p* < 0.0001). (**D**) Western blot analysis of LCM-BT474 cells after transfection with siBcl-3, siPIK, siSTAT3, or control siRNA (ctrl siRNA) for the proteins as indicated. (**A**,**D**) FastG (Fast green) or Comm. (Coomassie) staining was used to check for equal protein loading. NE = nuclear extract, CE = cytosolic extract, PM = plasma membrane extract.

To examine whether the effect of long-term CAF-CM treatment on Sox2 expression can also be observed with other ERα-positive breast cell lines, we generated the sublines LCM-BT474 and LCM-T47D from BT474 and T47D cell lines, respectively. Based on Western blot analysis, BT474 parental and LCM-BT474 cells express Sox2 at a similar well detectable level (Figure 7A). In contrast, Sox2 expression in T47D cells could not be visualized while it was faintly detectable in LCM-T47D. Hence, as judged by Western blot analysis, long-term treatment with CAF-CM increased overall Sox2 expression in T47D cells, but not in BT474 cells.

ICC analysis confirmed that Sox2 expression in BT474 is much higher than in T47D cells: the proportion of Sox2^hi^ cells in the BT474 cell line is 1.9% compared to 0.026% in the T47D cell lines, which is ~75× higher (Figure 7B,C). Additionally, in both cell lines, a subset of Sox2^low^ cells could clearly be distinguished from Sox2^hi^ cells and Sox2-negative cells. The proportion of Sox2^low^ cells is ~55× higher in BT474 compared to T47D (13% vs. 0.24%). Hence, in either cell line, the fraction of Sox2^low^ cells is 7–8-fold higher than the fraction of Sox2^hi^ cells. CAF-CM treatment increased the proportion of Sox2^low^ cells in both cell lines by ~3-fold in BT474 and by ~60-fold in T47D. Hence, Sox2^low^ cells represent ~37% of the LCM-BT474 and 2.8% of the LCM-T47D cell population. Additionally, CAF-CM increased the Sox2^hi^ cell population in T47D cells by ~60-fold, resulting in a proportion of Sox2^hi^ cells of 1.5% in the LCM-T47D subline. In sum, CAF-CM treatment strongly raised the number of Sox2-expressing cells also in the T47D and BT474 cell lines.

Examination of the importance of STAT3, Bcl-3, and AKT for Sox2 expression in the LCM-BT474 subline revealed that all three siRNAs, siSTAT3, siBcl3, and siPIK, reduced Sox2 expression, while the strongest effect was obtained with siSTAT3 (Figure 7D). Interestingly, siBcl3 and siPIK strongly decreased the P-STAT3 level in these cells. Hence, it is possible that the effects of siBcl3 and siPIK were at least partly mediated by STAT3. These data show that the same proteins that are responsible for Sox2 expression in LCM-AnD5 and LCMF-AnD5 are driving Sox2 expression in LCM-BT474.

Collectively, these data indicate that long-term exposure to CAF-CM raises the expression of Sox2 also in other ERα-positive breast cancer cell lines, involving the same proteins as in the MCF-7 cell line.

## 3. Discussion

The tumor microenvironment has a tremendous impact on the behavior of cancer cells. CAFs, which are part of the tumor microenvironment, are particularly important [17,58]. By secreting factors that alter cancer cell activities they contribute to tumor progression [48,59]. Here, the effect of the CAF-secretome (CAF-CM) on the transcription factor Sox2 in breast cancer cells was investigated. The rationale behind this study was the overwhelming evidence that this embryonal stem cell factor is involved in tumor growth, CSC activity and drug resistance [25,30,31,32,33,34,35,43,44,45,46]. Short-term as well as long-term effects of CAF-CM on MCF-7 breast cancer cells were examined. Experiments with prolonged exposure are important, as, in tumor patients, cancer cells are permanently in contact with their microenvironment. While short-term administration resulted in moderate upregulation of the Sox2 protein expression, long-term treatment yielded partly in huge Sox2 levels. Withdrawal of CAF-CM after long-term treatment had little effect on these high Sox2 level. This suggests that prolonged CAF-CM treatment resulted in permanently high Sox2 expression. While CAF-CM caused a rise in the Sox2 level in all cells of the MCF-7/AnD5 sublines and additionally increased the number of Sox2^hi^ cells, it affected only certain fractions of cells in the BT474 and T47D cell lines. In these cell lines, three types of Sox2-expresser could be distinguished, Sox2^hi^, Sox2^low^ cells, and cells with no Sox2 expression. In T47D, CAF-CM increased the proportion of both Sox2^hi^ and Sox2^low^ cells by ~60-fold, whereas, in BT474, it raised the proportion of only Sox2^low^ cells by ~3-fold. The percentage of cells expressing Sox2 after CAF-CM treatment rose to 37% in the BT474 and to 2.8% in the T47D population. Hence, in all three ERα-positive breast cancer cell lines studied, CAF-CM induced Sox2 expression, but to a different degree.

The observation that CAF-CM increased certain subpopulations of cells, Sox2^hi^ and Sox2^low^ cells, suggests that CAF-CM induced a selection process rather than upregulating expression of Sox2 in each individual cell. A selection process requires that the cell line studied is a heterogenous population. In fact, the MCF-7 cell line has been shown to contain different subclones, of which some are anti-estrogen resistant [60], while others show different morphology and spheroid formation ability [48]. Support of the notion that CAF-induced effects on protein expression is caused by selection comes from a previous study [59]. In this study, adaption of triple-negative breast MDA-MB-231 cancer cells to the CAF-secreted factors CXCL12 and IGF1 coincided with an increase in Src activity, which was then shown to be an result of an accumulation of pre-existing cells with a hyperactive Src pathway. In a similar way, adaption to CAF-CM could lead to a growth benefit of Sox2-expressing cells.

The growth benefit of Sox2-expressing cells in the presence of CAF-CM may be linked to the ability of Sox2 to suppress apoptosis (Figure 6D,E). Interestingly, gastric cancer cells respond to CAFs by showing a higher apoptotic activity [61]. We found a similar behavior of MCF-7 and AnD5 cells, which strongly increased PARP-1 fragmentation upon short-term treatment with CAF-CM (Figure S1H). Strikingly, short-term exposure to CAF-CM induced little or no PARP-1 fragmentation in Sox2-expressing LCM-AnD5, LCMF-AnD5, and C-FR1 cells. These findings raise the intriguing idea that, by inducing apoptosis, CAF-CM favors the growth of Sox2-expressing cells, which are better protected against apoptosis. As a consequence, these cells may also be more resistant to anti-estrogens, such as fulvestrant. Mechanistically, Sox2 may also increase fulvestrant resistance by inducing the expression of ABCG2 (ATP-binding cassette super-family G member 2) [44], a multidrug transporter protein [62]. This was shown for head and neck cell carcinoma cells, whose chemoresistant activity was caused by Sox2. However, in LCM-AnD5 and LCMF-AnD5 cells, knock-down of Sox2 did not alter the expression of ABCG2 (Figure 2). This suggests that ABCG2 is not involved in Sox2-induced fulvestrant resistance of CAF-CM-treated AnD5 cells.

Given the observation that siSox2 downregulated the expression of ERα (Figure 2), part of the effect of siSox2 on growth activity of LCM-AnD5 and LCMF-AnD5 cells may be indirectly caused by loss of this protein, as ERα inhibitor fulvestrant substantially reduced growth of both LCM-AnD5 and LCMF-AnD5 cell (Figure 6A–C). Both proliferation and survival of ERα-positive breast cancer cells have been shown to be dependent on ERα [63,64]. However, unlike siSox2, fulvestrant did not promote apoptosis. Nevertheless, it is still possible that the loss of ERα’s proliferative activity played a role in the siSox2 effect on cellular growth.

Since Sox2 expression in cancer has been linked to CSC activity [65], it raised the question of whether the Sox2-positive cells, particularly the Sox2^hi^ cells, in the CAF-CM-treated sublines are CSCs. The data show, that in the LCM-AnD5 and LCMF-AnD5 sublines, most Sox2^hi^ cells were not only found in CD44^hi^/CD24^lo^- and ALDH-positive subpopulations, but also in all other fractions, to the most part, at the same percentage. Only in the LCMF-AnD5 subline was the number of Sox2^hi^ cells increased in the ALDH-positive cell population, suggesting that a small fraction of Sox2^hi^ cells in this subline may be CSCs. On the other hand, Sox2 has been shown to increase ALDH1A1 expression [26]. Hence, they may as well be non-CSCs with Sox2-induced ALDH1A1 activity. Another support of the notion that most of the Sox2-positive cells in the CAF-CM-treated sublines are non-CSCs comes from the observation that siSox2 induced a strong decline in the level of ERα, which is primarily expressed in non-CSCs, as shown for the MCF-7 cell line as well as for ERα-positive primary breast cancer [22,66]. Altogether, these data suggest most Sox2-positive cells in these two sublines, irrespective of being high or low expresser, are non-CSCs. Nevertheless, CSCs might have contributed to the abundance of Sox2 in the non-CSCs by secreting Sox2-containing exosomes, as has been demonstrated for embryonic stem cells [67]. Another important point to be considered is that not all Sox2 expresser cells may be actually able to use Sox2 to run Sox2-dependent transcription, as studies with traceable Sox2 reporter plasmids on MCF-7 and gastric cancer cells have shown [32,46].

RNA interference studies revealed that STAT3, Bcl-3, and PI3K/AKT are important for maintaining the high Sox2 levels in the CAF-CM-treated sublines, as was shown for LCM-AnD5, LCMF-AnD5, and LCM-BT474. This is in line with a previous study with neural precursor cells, showing that STAT3 can directly bind to the Sox2 promoter and stimulate its activity [28]. STAT3 was also reported to mediate Sox2 expression in murine breast cancer cells in response to tumor-associated macrophages [29]. Bcl-3 has been shown to be important for Sox2 expression in murine embryonal stem cells [23] and AKT was found to drive Sox2 expression in hepatocellular carcinoma cells [27]. The impacts of the three factors on Sox2 expression seem to vary among the CAF-CM-treated sublines. STAT3 seems to be particularly important for LCM-AnD5 and LCM-BT474 cells, whereas Bcl-3 and PI3K/AKT are more critical in LCMF-AnD5 cells. In theory, the effects of Bcl-3 and AKT might be combined, as AKT is able to activate Bcl-3 directly by phosphorylating K33 [68] and to stabilize the Bcl-3 protein indirectly by preventing glycogen synthase kinase 3β-dependent phosphorylation at S394 and S398 [69]. *Vice versa*, in colorectal cancer cells, Bcl-3 was found to activate AKT [70]. In the LCM-AnD5 and LCMF-AnD5 cells, siBcl3 did not influence AKT phoshorylation (Figure 2). Nor did siPIK affected Bcl-3 expression. Hence, in these cells, Bcl-3 and PI3K/AKT seem to independently affect Sox2 expression. However, in CAF-CM-BT474 cells, siPIK reduced Bcl-3 expression (Figure 7D) suggesting that, in these cells, at least partly the PI3K/AKT pathway regulates Sox2 expression by affecting Bcl-3 expression. A link between Bcl-3 and STAT3 has also been reported [71,72], which may explain the downregulatory effect of siSTAT3 on Bcl-3 expression in LCM-AnD5 cells (Figure 2) and that of siBcl3 on the P-STAT3 level in LCM-BT474 cells (Figure 7D).

We have previously shown by using MCF-7 cells that Bcl-3 is important for fulvestrant resistance [18] and that the PI3K/AKT pathway acts as an anti-apoptotic pathway [19]. Hence, given the down-regulatory effect of siBcl3 and siPIK on Sox2 expression and given the anti-apoptotic and fulvestrant resistance-promoting effect of Sox2, Bcl-3, as well as AKT may have exerted their effects through Sox2.

A major component of CAF-CM is IL-6, which was found to share many effects of CAF-CM on protein expression, but was unable to induce fulvestrant resistance [19]. The likely reason for the latter was found be the failure of IL-6 to induce AKT phosphorylation. Here, we show that IL-6 is also unable to induce the expression of Sox2 and hence is not the mediator of the CAF-CM effect on Sox2. In one of the sublines (LIL6B-AnD5), Sox2 expression was even lower than in the parental subline AnD5 (Figure S1E). At a similar concentration, a downregulatory effect of IL-6 was also found on Sox2 expression in mesenchymal stem cells [73].

## 4. Materials and Methods

### 4.1. Cell Lines and Sublines

All cell lines and sublines were maintained in RMPI 1640 supplemented with 10% fetal calf serum (FCS) (PAN Biotech, Aidenbach, Germany) in the absence of antibiotics. The same batch of FCS was used for cell propagation and all experiments. All cell lines and sublines were authenticated by SNP analysis (Genolytic, Leipzig, Germany or Eurofins, Ebersberg, Germany). A summary of the sublines used is given in Table 1. Sublines LCM-AnD5, LCM-BT474 and LCM-T47D were established by propagating MCF-7/AnD5 [48], BT474 and T47D cells, respectively, in 20% CAF-CM. LCMF-AnD5 and LF-AnD5 cells were generated side-by-side by exposing AnD5 cells to 20% CAF-CM plus 1 nM fulvestrant (LKT Laboratories, St. Paul, MN, USA) or 1 nM fulvestrant alone, respectively. LIL6A-AnD5 and LIL6B-AnD5 sublines derived from AnD5 cells after incubation with 1 or 2.5 ng/mL rhIL-6 (PeproTech, Hamburg, Germany), respectively. During establishment (10 weeks) and for further propagation of the sublines, medium/serum and additives (CAF-CM, rhIL-6, fulvestrant) were replaced twice a week. All other sublines generated in our laboratory are previously described [48]. The tetracyclin-dependent CD44 expresser MCF-7 subline, MCF7/F-B5, was kindly provided by David Waugh [55]. To suppress CD44 expression, tetracyclin was added at a final concentration of 1 µg/mL.

Preparation of CAF-CM from 19TT human breast cancer-associated fibroblasts [74] was performed by collecting the medium and removing floating cells and debris by centrifugation at 3000 rpm for 15 min and filtration through a 0.45 µm-pore filter. For usage, one part of CAF-CM was mixed with four parts of fresh medium/serum (20% CAF-CM) before adding to breast cancer cell cultures.

### 4.2. Western Blot Analysis

Extraction of plasma membrane-bound, nuclear and cytosolic proteins and Western blot analysis were performed as previously described [48]. The primary antibodies used were as follows. Rabbit polyclonal antibodies: anti-P(S473)-AKT (1:2000, D9E, Cell Signaling Technology, Danvers, USA, MA), anti-Bcl-3 (1:1000, C-14, Santa Cruz Biotechnology, Heidelberg, Germany), anti-ERα (1:2000, HC-20, Santa Cruz Biotechnology, Heidelberg, Germany), anti-Sox2 (1:1000, D6D9, Cell Signaling Technology), anti-STAT3 (1:1000, 79D7, Cell Signaling Technology) and anti-P(Tyr705)-STAT3 (1:1000, D3A7, Cell Signaling Technology); rabbit monoclonal antibodies: anti-ABCG2 (1:1000, EPR20080, Abcam, Berlin, Germany), anti-FoxO3A (1:1000, D19A7, Cell Signaling Technology), anti-integrin β1 (1:2000, EPR1040Y, Abcam), anti-PARP-1, cleaved 25 kDa (1:10,000, Epitomics, Abcam) and anti-PI3 linase p110α (1:1000, C73F8, Cell Signaling Technology); mouse monoclonal antibodies: Anti-(pan)AKT (1:2000, 40D4, Cell Signaling Technology) and anti-CD44 (1:2000, Labvision, HCAM Ab-4, Life Technology, Darmstadt, Germany). Secondary antibody conjugates (anti-rabbit/anti-mouse horse radish peroxidase, 1:2000) were from Cell Signaling Technology. As antibodies against housekeeping proteins are no reliable markers for protein loading [75,76], protein loading was examined by either staining proteins with Fast Green (MERCK, Darmstadt, Germany) or with Coomassie Blue (Blue G, SERVA Electrophoresis, Heidelberg, Germany).

### 4.3. RNA Interferences

Cells were transfected by si (small interference) RNAs as described [77]. Briefly, following electroporation by using a Bio-Rad GenePulserX-cell (250 V, 800 μF), cells were seeded on a 10 cm culture dish and incubated for three days to allow the siRNA to downregulate its specific target. After that, cells were either lysed for protein extraction and Western blot analysis or trypsinized and further incubated for 4 days in 24-well plates for growth assays. For ICC analysis, transfected cells were incubated on slides (Superfrost, Menzel, Roth, Karlsruhe, Germany) for three days.

The sequences of siBcl3, siPIK, siSTAT3, siITGB1, and siLuc (control siRNA) are described elsewhere [18,19,48]. The sequences of siSox2 and siCD44 are as follows: siSox2 (sense: 5′-GGA CAG UUA CGC GCA CAU G-3′, antisense: 5′-CAU GUG CGC GUA ACU GUC C-3′), siCD44 (sense: CGA AUC CUG AAG ACA UCU A; antisense: UAG AUG UCU UCA GGA UUC G). All siRNAs were purchased from Eurofins.

### 4.4. Immunocytochemical Analyses (ICC)

For immunocytochemical analyses, cells were grown on Superfrost slides (Menzel), dried, fixed with methanol for 5 min, rehydrated in PBS/0.02% Tween 20 for 15 min, treated with 0.9% H_2_O_2_ for 10 min, rinsed with water, washed with PBS/0.02% Tween 20 for 5 min and incubated with an endogenous avidin/biotin blocking agent (Abcam, ab6421) by following the instructions of the manufacturer. After dilution of the primary antibody in Dako antibody diluent (anti-Sox2: 1:500, anti-PARP-1; cleaved 25 kDa: 1:5000), the slides were incubated with the antibody solution at 4 °C for overnight. Controls were run in Dako antibody diluent in the absence of the primary antibody. Detection of the primary antibody was performed by the ZytoChemPlus (horse radish peroxidase) Broad Spektrum Kit and AEC substrate Kit by following the instructions of the manufacturer (Zytomed, Berlin, Germany). For mounting and visualizing DNA, Immunoselect Antifading Mounting Medium DAPI (4′,6-diamidino-2-phenylindole) (Dianova, Hamburg, Germany) was used. For imaging, AxioCAM MRc5 camera and the AxioVision R 4.5 imaging software (version, Zeiss, Jena, Germany) was used.

### 4.5. Growth Assays

Growth activity was determined by an ATP/luciferase-based assay (Vialight Plus kit, Lonza, Cologne, Germany). In this assay, the ATP concentration, examined by luciferase activity, is used as a measure of the number of living cells. To assess growth activity of adherent cells, 10^4^ cells were added to each well of a 24-well plate and incubated for 4 days. After removal of the medium, cells were lysed by adding 100 μL PBS and 50 μL lysis buffer to each well. Then 50 μL of the cell lysate was mixed with 50 μL luciferase stock solution and the luciferase activity was measured by a Sirius luminometer (Berthold, Bad Wildbad, Germany).

### 4.6. FACS Analyses

Cells were detached by treatment with StemPro Accutase (Gibco, Life Technology, Darmstadt, Germany) for 5 min at 37 °C and cell aggregates removed by filtering the suspension through a 45 µm filter. Cells were centrifuged at 300× *g* for 5 min and resuspended in PBS supplemented with 0.5% FCS and 2 mM EDTA. After adjusting the cell concentration to 10^6^ cells/100 µL, 2 µL anti-CD24 APC-labeled antibody (Miltenyi Biotec, 130-112-846, Bergisch Gladbach, Germany) and 2 µL anti-CD44 PE-labeled antibody (Miltenyi Biotec, 130-113-904) were added, which was followed by incubation on ice for 10 min. Cells were then pelleted at 300 g for 5 min, washed with PBS/FCS/EDTA buffer, centrifuged again and resuspended in PBS/FCS/EDTA buffer (10^6^ cells/mL). The antibody-labeled cells were then separated by a BD FACSAria II sorter after normalizing for background fluorescence by using cells that were incubated in the PBS/FCS/EDTA buffer in the absence of antibodies. Cells of different fractions (CD44^hi^/CD24^lo^, CD44^hi^/CD24^hi^, and CD44^lo^/CD24^hi^) were directly sprayed on slides to examine their Sox2 expression by ICC.

For cell separation based on ALDH activity, the ALDEFLUOR kit (Stemcell Technologies, Cologne, Germany) was used. Cells were trypsinized, resuspended in RPMI/FCS, centrifuged and resuspended in ALDEFLUOR assay buffer (5 × 10^5^ cells/4 mL). After activating the ALDEFLUOR reagent by following the instruction of the manufacturer, 20 µL of activated ALDEFLUOR reagent was added to the cell suspension. To a second tube containing the same number of cells and the same reagents, 20 µL of the ALDH inhibitor DEAB was added. This control cell suspension served to normalize for background fluorescence. ALDH-positive and -negative cells were separated by a BD FACSAria II sorter and sprayed on slides to be examined for Sox2 expression by ICC.

### 4.7. Statistical Analyses

For parametric tests either the student’s *t*-test or one-way ANOVA combined with Bonferroni correction for post-hoc analysis was used. Non-parametric statistical analysis was done with the Mann-Whitney-U-test. A *p*-value of <0.05 was considered to indicate a statistically significant difference.

## 5. Conclusions

Our data suggest that prolonged interactions of ERα-positive breast cancer cells with secretory CAFs permanently increases the number of Sox2 expressing cells which to the most part are non-CSCs. This leads to a better protection against apoptosis and improves resistance to the anti-estrogen fulvestrant. STAT3, Bcl-3, and AKT were shown to strongly contribute to maintaining the high level of Sox2 expression in the long-term CAF-CM-treated sublines.

## Figures and Tables

**Table 1:** Description of the MCF-7 sublines used in this study.

Subline	Generated From	In the Presence of		Feature
		CM or rhIL-6	Fulvestrant	
C-FR1	MCF-7	CAF-CM	10^3^ nM	fulv res
C-FR2	MCF-7	CAF-CM	10^3^ nM	fulv res
C-FR3	MCF-7	CAF-CM	10^3^ nM	fulv res
M-FR1	MCF-7	MCF7-CM	10^3^ nM	fulv res
M-FR1	MCF-7	MCF7-CM	10^3^ nM	fulv res
AnD3 *	MCF-7	none	none	
AnD5 *	MCF-7	none	none	
AnD3/C-D1	AnD3 dormant cells	CAF-CM	10^3^ nM	
AnD3/C-D2	AnD3 dormant cells	CAF-CM	10^3^ nM	
AnD3/M-D1	AnD3 dormant cells	MCF7-CM	10^3^ nM	
AnD3/M-D2	AnD3 dormant cells	MCF7-CM	10^3^ nM	
AnD3/M-D3	AnD3 dormant cells	MCF7-CM	10^3^ nM	
LCM-AnD5	AnD5	CAF-CM	none	
LCMF-AnD5	AnD5	CAF-CM	1 nM	
LF-AnD5	AnD5	none	1 nM	
LIL6A-AnD5	AnD5	rhIL-6 (1.0 ng/mL)	none	
LIL6B-AnD5	AnD5	rhIL-6 (2.5 ng/mL)	none	
LCM-BT474	BT474	CAF-CM	none	
LCM-T47D	T47D	CAF-CM	none	

* AnD3 and AnD5 sublines are unable to generate fulvestrant-resistant cells in the presence of 103 nM fulvestrant and instead enter a dormant state, CM = conditioned medium, rhIL-6 = recombinant interleukin-6, fulv res = fulvestrant resistance.

## Supplementary Materials

The following are available online at https://www.mdpi.com/2072-6694/12/11/3435/s1, Figure S1 Additional data.
