# Supplementary material for: Carcinoma-Associated Fibroblasts Promote Growth of Sox2-Expressing Breast Cancer Cells
Source: Cancers (Basel). 2020 Nov 19;12(11):3435. doi: 10.3390/cancers12113435 (PMC7699386; doi:10.3390/cancers12113435)
Supplement: Supplementary file 1 [file cancers-12-03435-s001.pdf]

cancers

Carcinoma-Associated Fibroblasts Promote Growth of Sox2-Expressing Breast Cancer Cells

Angela Dittmer and Jürgen Dittmer

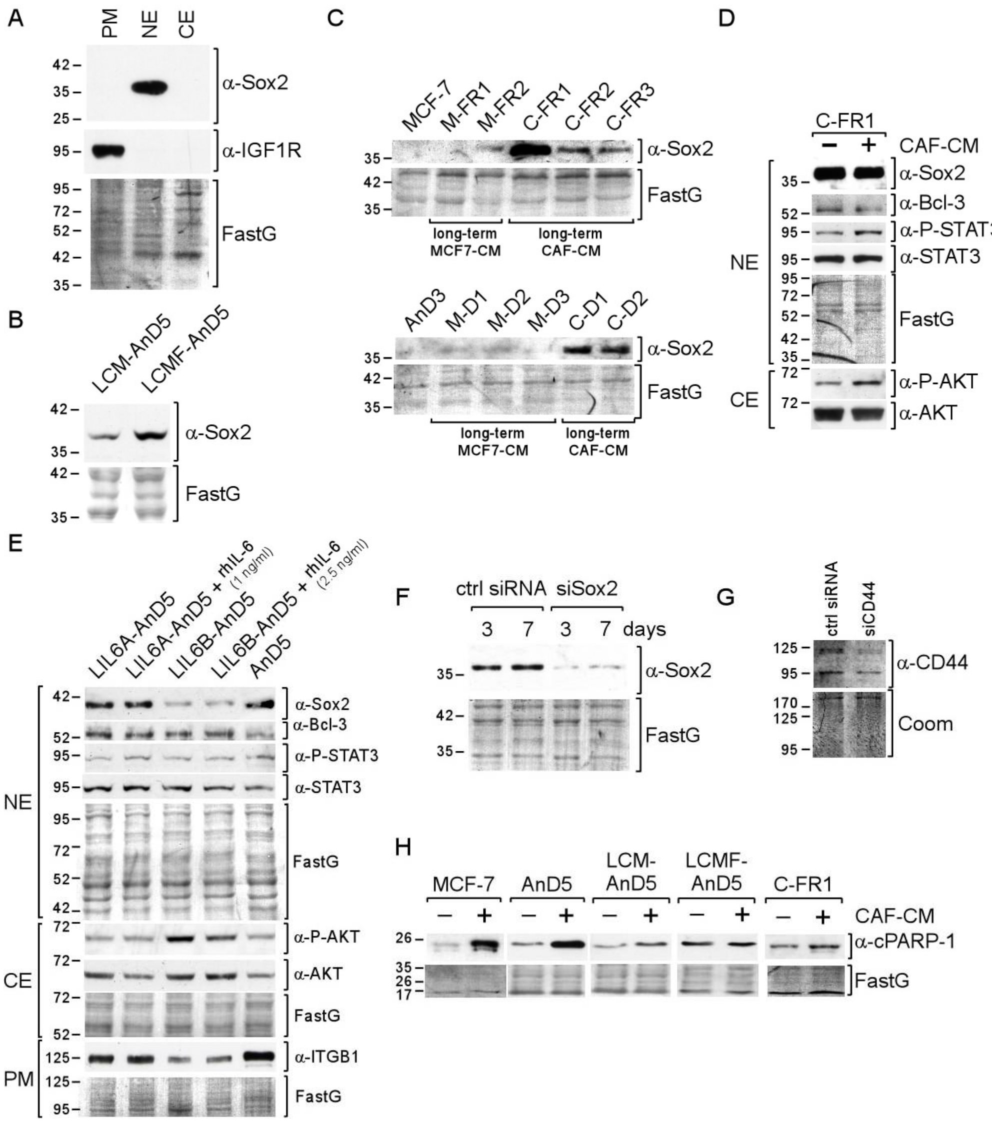

Figure S1. Western blot analyses of (**A**) different C-FR1 cell-derived protein extracts (PM = plasma membrane, NE = nuclear, CE = cytosolic) for Sox2 and IGF1R (insulin-like growth factor receptor 1), (**B**) nuclear extracts from LCM-AnD5 and LCMF-AnD cells for Sox2, (**C**) nuclear extracts of MCF-7 and MCF-7-derived fulvestrant resistant sublines M-FR1, M-FR2, C-FR1, C-FR2 and C-FR3 as well as MCF-7/AnD3-derived sublines M-D1, M-D2, M-D2, C-D1, C-D2 for Sox2, (**D**) C-FR1-derived protein extracts for the proteins as indicated, (**E**) protein extracts from rhIL-6 treated or untreated LIL6A-AnD5 and LIL6B-AnD5 cells as well as from AnD5 cells for the proteins as indicated, (**F**) nuclear extracts isolated from siSox2 or control (crtl) siRNA-transfected LCMF-AnD5 cells for Sox2, (**G**) plasma membrane extract from crtl siRNA- or siCD44-transfected LCM-AnD5 cells for CD44 and (**H**) cytosolic extracts from MCF-7, AnD5, LCM-AnD5, LCMF-AnD5 and C-FR1 cells for cPARP-1 in the presence or absence of CAF-CM (3 days). FastG = Fast Green. Coom = Coomassie.
